# Monash DaCRA fPET-fMRI: A dataset for comparison of radiotracer administration for high temporal resolution functional FDG-PET

**DOI:** 10.1093/gigascience/giac031

**Published:** 2022-04-30

**Authors:** Sharna D Jamadar, Emma X Liang, Shenjun Zhong, Phillip G D Ward, Alexandra Carey, Richard McIntyre, Zhaolin Chen, Gary F Egan

**Affiliations:** Monash Biomedical Imaging, Monash University, Melbourne, VIC 3800, Australia; Turner Institute for Brain and Mental Health, Monash University, Melbourne, VIC 3800, Australia; Australian Research Council Centre of Excellence for Integrative Brain Function, 3800 Australia; Monash Biomedical Imaging, Monash University, Melbourne, VIC 3800, Australia; Monash Biomedical Imaging, Monash University, Melbourne, VIC 3800, Australia; National Imaging Facility, 4072, Australia; Monash Biomedical Imaging, Monash University, Melbourne, VIC 3800, Australia; Australian Research Council Centre of Excellence for Integrative Brain Function, 3800 Australia; Monash Biomedical Imaging, Monash University, Melbourne, VIC 3800, Australia; Department of Medical Imaging, Monash Health, VIC 3800, Australia; Monash Biomedical Imaging, Monash University, Melbourne, VIC 3800, Australia; Department of Medical Imaging, Monash Health, VIC 3800, Australia; Monash Biomedical Imaging, Monash University, Melbourne, VIC 3800, Australia; Monash Data Futures Institute, Monash University , Melbourne, VIC 3800, Australia; Monash Biomedical Imaging, Monash University, Melbourne, VIC 3800, Australia; Turner Institute for Brain and Mental Health, Monash University, Melbourne, VIC 3800, Australia; Australian Research Council Centre of Excellence for Integrative Brain Function, 3800 Australia

**Keywords:** simultaneous PET/MR, functional PET, functional MRI, fluorodeoxyglucose positron emission tomography, blood oxygenation level–dependent functional magnetic resonance imaging, radiotracer administration, human neuroscience, human neuroimaging

## Abstract

**Background:**

“Functional” [^18^F]-fluorodeoxyglucose positron emission tomography (FDG-fPET) is a new approach for measuring glucose uptake in the human brain. The goal of FDG-fPET is to maintain a constant plasma supply of radioactive FDG in order to track, with high temporal resolution, the dynamic uptake of glucose during neuronal activity that occurs in response to a task or at rest. FDG-fPET has most often been applied in simultaneous BOLD-fMRI/FDG-fPET (blood oxygenation level–dependent functional MRI fluorodeoxyglucose functional positron emission tomography) imaging. BOLD-fMRI/FDG-fPET provides the capability to image the 2 primary sources of energetic dynamics in the brain, the cerebrovascular haemodynamic response and cerebral glucose uptake.

**Findings:**

In this Data Note, we describe an open access dataset, Monash DaCRA fPET-fMRI, which contrasts 3 radiotracer administration protocols for FDG-fPET: bolus, constant infusion, and hybrid bolus/infusion. Participants (n = 5 in each group) were randomly assigned to each radiotracer administration protocol and underwent simultaneous BOLD-fMRI/FDG-fPET scanning while viewing a flickering checkerboard. The bolus group received the full FDG dose in a standard bolus administration, the infusion group received the full FDG dose as a slow infusion over the duration of the scan, and the bolus-infusion group received 50% of the FDG dose as bolus and 50% as constant infusion. We validate the dataset by contrasting plasma radioactivity, grey matter mean uptake, and task-related activity in the visual cortex.

**Conclusions:**

The Monash DaCRA fPET-fMRI dataset provides significant reuse value for researchers interested in the comparison of signal dynamics in fPET, and its relationship with fMRI task-evoked activity.

## Background

The neural functions of the human brain rely upon a stable and reliable energy supply delivered in the form of glucose [1]. The human brain accounts for 20% of the body's energy consumption at rest [2, 3], of which 70–80% is used by neurons during synaptic transmission. Global and regional variations in the glucose uptake during neural activity can be measured using the [18]-fluorodeoxyglucose positron emission tomography (FDG-PET) method. Because cerebral glucose uptake primarily reflects synaptic transmission [2], FDG-PET has long been used in neuroimaging studies as a proxy for neuronal activity. In recent years, functional brain imaging studies using the FDG-PET method have been somewhat overshadowed by the blood oxygenation level–dependent functional magnetic resonance imaging (BOLD-fMRI) method. This is primarily due to the improved spatial and temporal resolution of fMRI in comparison to FDG-PET. Traditional FDG-PET methods provided a snapshot of glucose uptake averaged across the uptake and scan periods (duration ∼30 mins) and were unable to distinguish between neural responses to stimuli presented closely in time. However, the recent availability of molecular MRI scanners, which provide the capacity to simultaneously acquire BOLD-fMRI and FDG-PET data, has driven significant advances in FDG-PET methodologies for human neuroscience functional brain mapping studies [4–7].

Recently, improvements in radiotracer delivery have resulted in substantial improvement in the temporal resolution of FDG-PET. The method described as “functional” PET (fPET) involves delivering the radiotracer as a constant infusion over the course of the scan. In a landmark study, Villien et al. [7] adapted the constant-infusion technique [8] to deliver sufficient radiotracer to measure dynamic changes in brain glucose metabolism in response to a checkerboard stimulation, with a temporal resolution of 1 minute. Using fPET data acquired simultaneously with (non-functional) MRI (i.e., MRI/fPET), Villien et al. were able to estimate a general linear model (GLM) response for blocked stimuli presented 5–10 mins apart. Subsequent studies have extended these findings and achieved fPET temporal resolutions of 1 min [6, 7, 9–11] or less (12 sec [5]; 16 sec [12–14]; 30 sec [15]).

The PET image quality relies upon the neural tissue radioactivity count rate from the administered radiotracer and the duration of the scan [16]. fPET protocols typically have lower signal-to-noise ratio than static FDG-PET acquisitions because the constant-infusion approach must administer the same effective dose of radioactivity over a longer period. Furthermore, fPET protocols require commencement of the scanning to be synchronized with the start of the radiotracer administration to ensure that the measured brain activity is specific to the activity evoked during the experiment. Consequently, a constant-infusion fPET scan has very little (close to zero) signal at the commencement of the experimental protocol, and the signal continuously increases over the duration of the infusion and scan [5, 12]. Constant-infusion fPET imaging protocols therefore tend to be quite long in comparison to standard FDG-PET and fMRI neuroimaging studies—usually ∼90–100 mins [6, 15]. These considerations restrict fPET studies primarily to populations that are able to comply with scanning requirements (e.g., restricted movement) over a long period.

The aim in acquiring the Monash Dataset for Comparison of Radiotracer Administration fPET-fMRI (“Monash DaCRA fPET-fMRI”) [17] was to contrast different radiotracer administration protocols for fPET data acquisition. The majority of fPET studies have used a constant-infusion delivery protocol [6, 7, 9–11, 14], where the entire dose of radiation is provided as an infusion over the course of the scan. However, a small number of studies have examined whether a hybrid bolus plus infusion protocol (bolus-infusion) might provide better signal at early timepoints while still allowing task-related activity to be measured at later timepoints. In a proof-of-concept comparison, we [12] found that a bolus-infusion protocol—where 50% of the dose was delivered as bolus, 50% as infusion—seemed to provide the most stable fPET signal for the longest period, compared with 100% constant infusion or 100% bolus protocols. Note, however, that this result was obtained in a case study design. Rischka et al. [5] used a 20% bolus plus 80% infusion protocol to test the lowest task duration detectable with fPET methodology. They were able to measure task-related activity (finger tapping) to stimuli separated by 2 mins with an fPET frame size of 12 sec using this protocol; no signal was detected for stimuli separated by 1 min with 6-sec PET frame size. Rischka et al. concluded that the bolus-infusion protocol allowed assessment of reduced duration task blocks. However, they did not compare bolus-infusion with either constant infusion or bolus administration. In a subsequent study from the same group, Riscka et al. [18] demonstrated excellent reliability of fPET at rest with 20% bolus 80% infusion administration; reliability of fPET reduced during task performance; and lowest reliability for BOLD-fMRI during rest and task.

**Figure 1:**
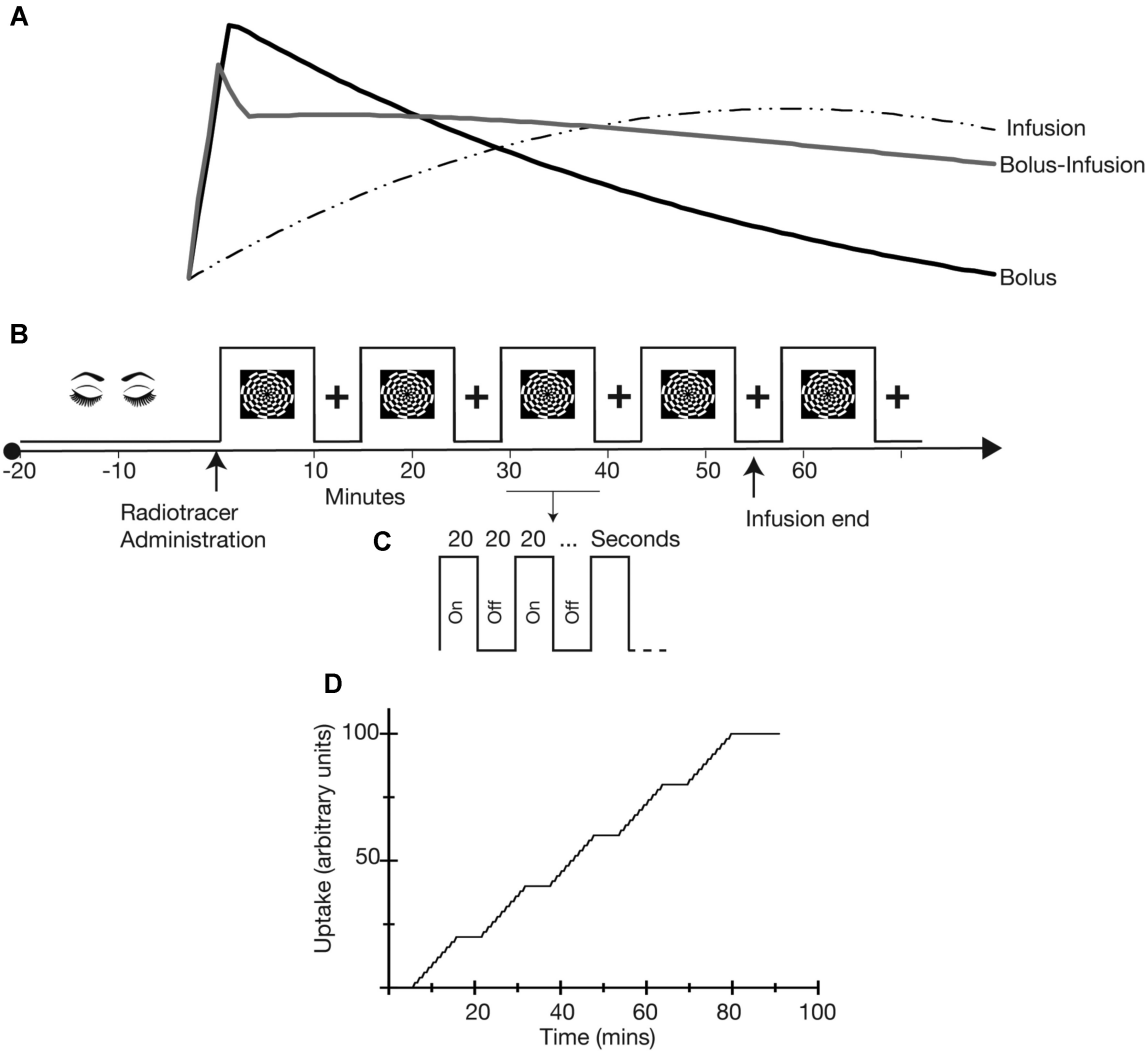
**A**. Hypothesized plasma radioactivity curves for the 3 administration protocols. Timing (i.e., signal peak and duration) is shown in comparison to the timing of the experimental protocol shown in panel B. We hypothesized that the bolus protocol would peak soon after administration and decline rather quickly thereafter, returning to baseline levels by the end of the scan. We predicted that the bolus protocol would show the largest overall peak signal. For the infusion protocol we hypothesized that radioactivity would be close to zero at the beginning of the scan, continuing to increase for the duration of the scan. For the bolus-infusion protocol, we predicted that the peak signal would occur around the same time as the bolus protocol but be of smaller magnitude. We expected the signal would decrease slightly but then remain at elevated levels for the duration of the scan. **B, C**. Experimental protocol. Checkerboard stimuli were presented in an embedded block design, with fast on/off periods (panel C) embedded within the longer “on” (panel B) periods. **D**. Predicted task-related timecourse for the fPET general linear model.

Here, we acquired fPET data with 50/50 bolus-infusion, 100% constant infusion, and 100% bolus protocols. We chose to start with a proportional 50/50 bolus/infusion protocol rather than some other fraction (e.g., 20/80) as a starting point for parsimony. Figure 1A illustrates our expectations for the fPET signal for the 3 protocols. Consistent with the results from our proof-of-concept case study, we expected that the bolus protocol would provide the largest overall signal magnitude, with the peak early in the scan period, decreasing in magnitude across the duration of the scan. The fPET signal for the constant-infusion protocol was predicted to increase slowly through the course of the scan, with the overall lowest peak magnitude. Last, the fPET signal for the hybrid bolus-infusion protocol was expected to show the overall longest sustained period over the course of the scan. We predicted that the bolus-infusion protocol would provide the best sensitivity for detecting task-related effects in the checkerboard stimulus task, followed by the constant infusion then bolus protocol.

We present 1 approach for GLM-based analysis of fPET data for data validation and quality control, and as an example of the type of analyses that are possible with this dataset. Development of more sophisticated methods of GLM and ICA analyses are examples of potential reuses of the dataset.

## Methods

All methods were reviewed by the Monash University Human Research Ethics Committee, in accordance with the Australian National Statement on Ethical Conduct in Human Research (2007). Administration of ionizing radiation was approved by the Monash Health Principal Medical Physicist, in accordance with the Australian Radiation Protection and Nuclear Safety Agency Code of Practice (2005). For participants older than 18 years, the annual radiation exposure limit of 5 mSv applies; the effective dose in this study was 4.9 mSv. Detailed information on the method for acquiring fPET data using bolus, constant-infusion, and bolus-infusion protocols is reported in Jamadar et al. [12].

### Available data

Data are available on OpenNeuro with the accession No. ds003397 [17].

The data (Table 1) include participant information (demographic characteristics), scan information (e.g., start times), blood information (plasma radioactivity), raw MRI data (T1, T2 FLAIR, MR attenuation correction, susceptibility weighted images, field maps), unreconstructed PET data, and reconstructed PET images with temporal bins of 16 sec. Plasma radioactivity for 1 participant (participant 14) was incomplete because blood could not be drawn after the third timepoint.

**Table 1:** Data fields for the Monash radfPET-fMRI dataset

Data	Field	Type
participants.tsv	Participant ID	
	Group	Categorical: B: bolus, I: infusion, B/I: bolus/infusion
	Haemoglobin (Hb)	Numeric
	Blood sugar level (BSL)	Numeric
	Age	Numeric
	Gender	String
	Years of education	Numeric
	Highest level education completed	Categorical: 1. No formal education; 2. Primary school (year 6); 3. High school (year 10); 4. High school (year 12); 5. Trade certificate; 6. Bachelors degree; 7. Postgraduate (Masters); 8. Ph.D. or Doctorate
	English as first language	Yes/no
	Visual impairment	Yes/no; self-reported
	Visual impairment—specify	String
	Hearing impairment	Yes/no; self-reported
	Hearing—specify	String
	Personal history mental illness	Yes/no
	Personal history mental illness—specify	String
	Personal history mental illness—ongoing	Yes/no
	Family history mental illness	Yes/no
	Family history mental illness—specify	String
	Family history dementia	Yes/no
	Family history dementia—specify	String
	Family history dementia—ever diagnosed	Yes/no
	Cardiovascular illness—ever	Yes/no
	Diabetes—ever	Yes/no
	Current tobacco	Yes/no
	Current tobacco average; how many per day?	String
	Ever smoked tobacco	Yes/no
	Ever smoked tobacco average; how many per day?	String
	Ever consumed alcohol	Yes/no
	Alcohol—how often	String
	Standard drinks per drinking occasion	String
	Recreational drugs last 6 months	Yes/no
	Recreational drugs—specify	String
	Recreational drugs—how often	String
dose.tsv	Participant ID	
	Group	Categorical: B: bolus, I: infusion, B/I: bolus/infusion
	Actual dose—bolus	MBq
	Actual dose—infusion	MBq
	Total infusion duration	hh:mm:ss
	PET start time	Clock time; hh:mm:ss
	Bolus start time	Clock time; hh:mm:ss
	Infusion start time	Clock time; hh:mm:ss
	Echo planar imaging (EPI) start time	Clock time; hh:mm:ss
bloods.tsv	Participant ID	
	Time sample was taken	Clock time; hh:mm:ss
	Time measurement of radioactivity was taken	Clock time; hh:mm:ss
	Counts per minute for each timepoint	Numeric, multiple entries per timepoint, 0–10
	Total counts for each timepoint	Numeric, multiple entries per timepoint, 0–10
sub_*	BIDS dataset	
anat	T1 weighted image data	Data in NIfTI format and metadata in JSON sidecar
fmap	Functional maps in magnitude and phase	Data in NIfTI format and metadata in JSON sidecar
func	Functional MRI data	Data in NIfTI format and metadata in JSON sidecar
pet	Reconstructed PET data with 16-sec bins	Data in NIfTI format and metadata in JSON sidecar
ute	UTE scans	Data in NIfTI format and metadata in JSON sidecar
dixon	Dixon scans	Data in NIfTI format and metadata in JSON sidecar
sourcedata/sub_*		
pet/*_listmode*	Raw listmode PET data	Data in binary format and metadata in JSON sidecar
pet/*_norm*	PET normalization data	Data in binary format and metadata in JSON sidecar
pet/*_sinogram*	PET sinogram data	Data in binary format and metadata in JSON sidecar
pet/*_physio*	PET physio data	Data in binary format and metadata in JSON sidecar
pet/*_recording-blood_discrete*	Blood plasma measurement data	tsv tabular format and metadata in *_blood.json sidecar

### Participants

Fifteen young adults participated in this study. Participants were randomly assigned to the bolus, infusion, and bolus-infusion groups. Participants were aged 18–20 years, right handed, had normal or corrected-to-normal vision, and were screened for diabetes, hearing impairment, personal or family history of mental or neurodegenerative illness, and personal history of head injury or neurological condition. Women were screened for pregnancy. Prior to the scan, participants were directed to consume a high-protein/low-sugar diet for 24 hours, fast for 6 hours, and drink 2–6 glasses of water.

Participants in the bolus group had mean age 19.2 years, 3 were male, and had 12–14 years of education (mean 13.2 years). Participants in the infusion group had mean age 19.4 years, 4 were male, and had 14–15 years of education (mean 14.6 years). Participants in the bolus-infusion group had mean age 19.4 years, 1 was male, and had 12–15 years of education (mean 13.6 years).

### Stimuli and tasks

Participants rested with eyes closed during the initial 20 mins while non-functional MR scans were acquired. During simultaneous fMRI-fPET scanning, participants viewed flickering checkerboard stimuli presented in an embedded block design [6]. We have previously shown that an embedded design provides simultaneous contrast for task-evoked BOLD-fMRI and FDG-fPET data. The task alternates between 640-sec flashing checkerboard blocks and 320-sec rest blocks (Fig. 1B). This slow alternation provides fPET contrast. Within the 640-sec checkerboard blocks, checkerboard and rest period alternate with a rate of 20 sec on, 20 sec off (Fig. 1C). This fast alternation is suitable for BOLD-fMRI contrast.

The checkerboard stimulus was a circular checkerboard of size 39 cm (visual angle 9°) presented on a black background. The checkerboard flickered (i.e., alternated black and white segments) at 8 Hz. During the “off” periods, participants rested with eyes fixated on a white cross of size 3 cm (visual angle 0°45′).

### Procedure

Participants were cannulated in the vein in each forearm with a minimum size 22-gauge cannula. A 10-mL baseline blood sample was taken at time of cannulation. For all participants, the left cannula was used for FDG administration, and the right cannula was used for blood sampling. Primed extension tubing was connected to the right cannula for blood sampling via a 3-way tap.

Participants underwent a 95-min simultaneous MRI-PET scan in a Siemens Biograph 3Tesla molecular MR (mMR) scanner. Participants lay supine in the scanner bore with head in a 16-channel radiofrequency head coil and were instructed to lie as still as possible. [^18^F]-FDG (mean dose = 238 MBq) was administered either as a bolus, an infusion, or as a bolus-infusion (50% bolus 50% infusion). For the infusion protocols, infusion rate was 36 mL/hour using a BodyGuard 323 MR-compatible infusion pump (Caesarea Medical Electronics, Caesarea, Israel). For the bolus protocol, the bolus was administered at the time of the PET scan onset. For the infusion protocol, the infusion commenced at the time of PET scan onset. For the bolus-infusion protocol, the bolus was administered at the onset time of the PET scan, and the infusion started as soon as possible (mean = 40 sec) after the bolus. For the infusion and bolus-infusion protocols, the infusion ceased at 55 mins. We hypothesized that the plasma radioactivity would be maintained for a short period thereafter; however this was not the case (see Data Validation Results - Plasma radioactivity).

Plasma radioactivity levels were measured throughout the duration of the scan. At 5 mins after administration, a 10-mL blood sample was taken from the right forearm using a vacutainer; the time of the 5-mL mark was noted for subsequent decay correction. Subsequent blood samples were taken at 5-min intervals. The cannula line was flushed with 10 mL of saline after every sample to minimize line clotting. Immediately after sampling, the sample was placed in a Hereaus Megafuge 16 centrifuge (ThermoFisher Scientific, Osterode, Germany) and spun at 2,000 rpm for 5 mins; a 1,000-μL quantity was pipetted, transferred to a counting tube, and placed in a well counter for 4 mins. The count start time, total number of counts, and counts per minute were recorded for each sample.

### MR-PET protocol

PET data (90:56 min) were acquired in list mode. The onset of the PET acquisition (and the radiotracer administration) was locked to the onset of the T2* echo-planar images (EPIs).

The MRI and PET scans were acquired in the following order: (i) T1-weighted 3D MPRAGE (TA = 7.01 min, TR = 1,640 ms, TE = 2.34 ms, flip angle = 8°, field of view [FOV] = 256 × 256 mm^2^, voxel size = 1 × 1 × 1 mm^3^, 176 slices, sagittal acquisition); (ii) T2-weighted FLAIR (TA = 5.78 mins); (iii) SWI (TA = 6.83 mins); (iv) gradient field map (TA = 1.08 mins); (v) MR attenuation correction Dixon (TA = 0.65 mins, TR = 4.14 ms, TE in phase = 2.51 ms, TE out phase = 1.3 ms, flip angle = 10°); (vi) T2*-weighted EPIs (TA = 90:56 mins; TR = 4,000 ms, TE = 30 ms, FOV = 190 mm, 3 × 3 × 3 mm voxels, 44 slices, ascending axial acquisition), P-A phase correction (TA = 0.37 mins); (vii) UTE (TA = 1.97 mins).

## Data Records

Detailed information about the data records available for the Monash DaCRA fPET-fMRI dataset (OpenNeuro ds003397) [17] is reported in Table 1. Table 2 reports the software used in this article.

**Table 2:** : Software used in the development of this article

Project name	Project home page	Version	Operating system	Programming language	Other requirements	License
mrpet-bids	[19]	v1.0	Linux, Unix	Python	Python 3.5+	Apache-2.0
heudiconv	[20]	0.8.0	Linux, Unix	Python	Python 3.x	Apache-2.0
dcm2niix	[21]	1.0.20200427	Platform independent	C/C++		BSD
pydeface	[22]	2.0.0	Platform independent	Python		MIT
ANTs	[23]	v2.3.4	Linux, Mac, Windows	C/C++, Shell		Copyright (c) 2009–2013 ConsortiumOfANTS
FSL	[24]	6.0.3	Platform independent	C/C++		GPLv2
AFNI	[25]	21.2.03	Linux, MacOS	C/C++		GPL

Participants.tsv is a text file reporting demographic and anthropometric data for each participant, ordered by participant ID. Plasma_radioactivity.tsv is a text file reporting the plasma radioactivity counts and measurement times for each participant, ordered by participant ID.

The dataset contains both raw (unprocessed) images and source data (i.e., unreconstructed PET listmode data). Both are organized in subdirectories that correspond to participant ID, according to BIDS (for MRI) or BIDS-consistent (for PET) specification. For each participant, T1-weighted MPRAGE images, fMRI images, and gradient field maps are in the “anat” (anatomical data), “func” (functional MRI data), and “fmap” (field map) subdirectories, along with metadata in the json sidecar. Dixon and UTE scans are available for PET source data reconstruction, which are organized into “dixon” and “ute” subdirectories.

Although there is not currently a listmode PET BIDS specification, the same structure is followed with a json sidecar accompanying the image data. PET image data were obtained by reconstructing the PET source data into 16-sec bins offline using Siemens Syngo E11p. Attenuation was corrected using pseudoCT [26] Ordinary Poisson-Ordered Subset Expectation Maximization (OP-OSEM) algorithm with point-spread function modelling [27] with 3 iterations, 21 subsets, and 344 × 344 × 127 (voxel size = 2.09 × 2.09 × 2.03 mm^3^) reconstruction matrix size. A 5-mm 3D Gaussian post-filtering was applied to the final reconstructed images. Following the BIDS extension for PET (BEP009), blood data are also included in the “pet” directory, which report the plasma radioactivity counts and measurement times for the participant. Data in sub-^∗^/dixon and sub-^∗^/ute are ignored in the BIDS validation process because they are not officially supported by the current BIDS specification.

The “sourcedata” directory contains the raw, un-reconstructed PET source data that were directly exported from the Siemens scanner console. The source data include PET listmode data, normalization data, sinogram data, and physiology data. The raw PET data are in the form of a file pair (1 DICOM header and 1 binary file) with the 2 paired files having the same file name but different extensions (.dcm for DICOM, .bf for binary). A json metadata sidecar file was added to each participant's raw dataset, consistent with the BIDS approach for supported structures. The blood plasma radioactivity data is included and is identical to the reconstructed PET image data. The sourcedata directory is also excluded in the BIDS validation process.

To prepare the BIDS dataset, the open source conversion tool Heudiconv [20] was used to organize the imaging data into structured directory layouts, and the dcm2niix converter [21] was used to convert image data from dicom to NIfTI format. Following the approach in our previous article [28], we applied scripts to (i) remove personal identifiable information from the raw PET dicom header, (ii) add custom json sidecar files to the PET raw data and reconstructed image data, and (iii) generate plasma radioactivity files. Refer to [19] for these scripts.

Defacing was applied to T1-weighted, images using pydeface [22]. Reconstructed PET images and PET raw data were not defaced because participants cannot be visually identified from the PET images.

## Data Validation—Methods

We validated the Monash DaCRA fPET-fMRI dataset by confirming that the data yielded expected results with standard GLM analysis.

### fMRI image preparation and analysis

The participants' T1 brain images were extracted (ANTs [29]), to standard space using affine transformation (12 degrees of freedom) and a standard space 2-mm brain atlas. The T1 image was corrected for intensity nonuniformity using N4 Bias field correction (ANTs [30]), segmented using FSL following the routine of Parkes et al. [31] then normalized to MNI152 space. The EPI scans for all participants underwent a standard fMRI pre-processing pipeline. All EPI scans were brain extracted (FSL BET [32]), motion corrected (FSL MCFLIRT [33]), and slice timing corrected (AFNI).

Pre-processed fMRI data were submitted to a participant-level GLM using FSL [34] FEAT. The following pre-statistics were applied: spatial smoothing using a Gaussian kernel of full width at half-maximum (FWHM) 5 mm; grand-mean intensity normalization of the entire 4D dataset by a single multiplicative factor; and high-pass temporal filtering (Gaussian-weighted least-squares straight line fitting, with sigma = 50.0s). Time-series statistical analysis was carried out using FILM with local autocorrelation correction [35]. For the participant-level analysis we used a GLM where the only regressor of interest was task, and temporal derivative as covariate. Participant-level *Z* (Gaussianized) static images were thresholded non-parametrically using clusters determined by *Z* > 1.6 and a corrected cluster significance threshold of *P* = 0.05 [36]. Group-level analysis was carried out using FLAME (FMRIB's Local Analysis of Mixed Effects) stage 1 [37–39] to obtain the group mean. Three separate group-level GLMs were conducted for each group (bolus, infusion, bolus-infusion).

### PET image preparation and analysis

Spatial realignment was performed on the dynamic FDG-fPET images using FSL MCFLIRT [33]. A mean FDG-PET image was derived from the entire dynamic time series and rigidly normalized to the individual's high-resolution T1-weighted image using ANTs [29]. The dynamic FDG-fPET images were then normalized to MNI space using the rigid transform in combination with the non-linear T1 to MNI warp. fPET images were spatially smoothed using a Gaussian kernel of 12-mm FWHM. The mean of fPET signal across the entire grey matter mask was estimated and included in subsequent analysis.

fPET data processing was carried out using FEAT (fMRI Expert Analysis Tool) version 6.00. The pre-processed smoothed MNI152-space fPET images were submitted to a GLM analysis using FILM [35].

We modelled the increasing whole-brain radioactivity signal related to radiotracer uptake across the PET scan period. For each participant, we assume an underlying baseline activity (Y_base_) when no task was performed to model the radiotracer uptake. We subtracted Y_base_ from the time-series data to obtain baseline-corrected data:

$$\begin{eqnarray*}
{{\rm{Y}}_{{\rm{basecorr}}}} = {\rm{Y}} - {{\rm{Y}}_{{\rm{base}}}}
\end{eqnarray*}
$$

where Y_base_ is the underlying baseline time series for each voxel.

Then, we can estimate β_task_ as

$$\begin{eqnarray*}
{\rm Y}_{\rm basecorr} = {\beta_{\rm task\,\cdot regressor_{\rm task}}} + {\rm \varepsilon }.
\end{eqnarray*}
$$

In another GLM, we approximate the baseline using grey matter mean (regressor_GM_) as confound:

$$\begin{eqnarray*}
{\rm Y} = {\beta ^{\prime}_{\rm task \cdot\, regressor}}_{\rm task} + {\beta_{\rm GM\,regressor}}_{\rm GM} + {\rm \varepsilon^{\prime}}
\end{eqnarray*}
$$

Thus, the “cleaned” data are represented as

$$\begin{eqnarray*}
\
{{\rm Y}_{\rm clean}} = {\rm Y} - {\beta_{{\rm GM\,\, regressor}_{\rm GM}}} = {\beta ^{\prime}_{{\rm task\,\,\cdot regressor}}}_{\rm task} + {\rm \varepsilon ^{\prime}}
\end{eqnarray*}
$$

also

$$\begin{eqnarray*}
{\rm Y} = {\rm Y}_{\rm clean} + {\beta _{\rm GM\,\,regressor}}_{\rm GM}
\end{eqnarray*}
$$

Because

$$\begin{eqnarray*}
{{\rm{Y}}_{{\rm{basecorr}}}} = {\rm{Y}} - {{\rm{Y}}_{{\rm{base}}}},
\end{eqnarray*}
$$

then,

$$\begin{eqnarray*}
{\rm Y}_{\rm basecorr} = {\rm Y}_{\rm clean} + {\beta_{\rm GM\,\,regressor}}_{\rm GM} - {\rm Y}_{\rm base} = {\beta_{\rm task\,\,\cdot\,\,regressor}}_{\rm task} + {\rm \varepsilon}
\end{eqnarray*}
$$

i.e.,

$$\begin{eqnarray*}
{\rm Y}_{\rm clean} = {\beta_{\rm task\,\,\cdot\,\,regressor}}_{\rm task} + {\rm Y}_{\rm base} - {\beta _{\rm GM\,\,regressor}}_{\rm GM} + {\rm \varepsilon}
\end{eqnarray*}
$$

Because the baseline Y_base_ coefficient of 1 can be expressed as

$$\begin{eqnarray*}
1 = k\cdot{\beta _{{\rm{GM}}}}
\end{eqnarray*}
$$

then

$$\begin{eqnarray*}
{\rm Y}_{\rm clean} = {\beta _{\rm task\,\,\cdot\,\,regressor}}_{\rm task} + (k\cdot{\beta _{\rm GM}}\cdot{\rm Y}_{\rm base} - {\beta_{\rm GM\,\,regressor}}_{\rm GM}) + {\rm \varepsilon}
\end{eqnarray*}
$$

$$\begin{eqnarray*}
{\rm Y}_{\rm clean} = {\beta _{\rm task\,\,\cdot\,\,regressor}}_{\rm task} + {\beta _{\rm GM}(k\cdot{{\rm Y}_{\rm base}} - {\rm regressor}_{\rm GM}) + {\rm{\varepsilon }}}
\end{eqnarray*}
$$

**Figure 2:**
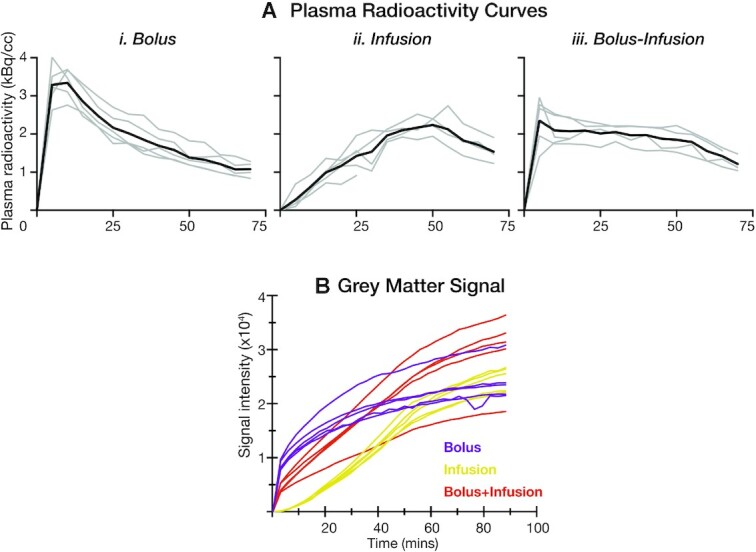
**A**. Plasma radioactivity curves (decay corrected) for (**i)** bolus administration, (**ii)** infusion administration, and (**iii)** bolus-infusion protocol. Black line shows mean radioactivity and grey lines show activity for individual participants. **B**. Mean grey matter signal across all voxels for each participant, calculated from reconstructed PET images prior to GLM analysis. Because the images are not quantified, units are arbitrary intensity. Grey lines indicate approximate area of task blocks (also refer to Fig. 1).

For the 3 tracer administration protocols, the uptake trends in time activity curve (TAC) are very different (see our data in Fig. 2B), which complicates the comparison between the 3 groups. To simplify the comparison between groups, we assume that the whole-brain grey matter mean over the scanning period is in a similar uptake trend with baseline uptake for each voxel, and only consider the linear term of the difference between each ROI and the grey matter mean. We therefore used a linear change *n* · regressor_line_ to approximately replace (*k* · Y_base_ − regressor_GM_).

Then

$$\begin{eqnarray*}
{\rm Y}_{\rm clean} = {\beta_{\rm task\,\,\cdot\,\,regressor}}_{\rm task} + {\beta _{\rm line\,\,\cdot\,\,regressor}}_{\rm line} + {\rm{\varepsilon }}
\end{eqnarray*}
$$

where

$$\begin{eqnarray*}
{\beta _{{\rm{line}}}} = n\cdot{\beta _{{\rm{GM}}}}
\end{eqnarray*}
$$

The participant-level GLM had 2 regressors: namely, a task regressor (Fig. 1D) and a linear regressor that modelled the continuous underlying baseline uptake over time.

Participant-level *Z* (Gaussianized) static images were thresholded non-parametrically using clusters determined by *Z* > 1.6 and a corrected cluster significance threshold of *P* = 0.05 (FWE corrected) [36]. Percent signal change was calculated across all task blocks relative to rest blocks using FSL Featquery. Group-level analysis was carried out using FLAME stage 1 [37–39] to obtain the group mean activation map. Because the baseline uptake rate differed throughout the brain, in some voxels the baseline regressed time-series data showed a negative trend because the uptake rate was lower than the grey matter mean uptake rate. To determine the brain regions that associated negatively with the task we included a contrast to model negative task events.

## Data Validation—Results

### Plasma radioactivity

We hypothesized shapes of the radioactivity curves for the 3 groups assuming that the bolus-infusion protocol would provide the best sensitivity for detecting task-related effects in the checkerboard stimulus task, followed by the constant infusion and the bolus protocol (Fig. 1A). The measured plasma radioactivity curves for the 3 radiotracer administration protocols are shown in Fig. 2A. Radioactivity peaked early and declined quickly for the bolus protocol. The largest radioactivity peak was evident in the bolus protocol. In the infusion protocol, radioactivity continued to increase until the cessation of the infusion (55 mins), at which point activity declined. The continued upward slope of the curve for the duration of the infusion suggests that the plasma radioactivity had not yet reached its peak before the cessation of the infusion. As predicted, the bolus-infusion protocol showed an early peak after the bolus; the activity decreased slightly but was maintained at close to a constant level for the duration of the infusion. As expected, the peak for the bolus-infusion protocol was smaller than in the bolus protocol.

As noted in the Methods, for the infusion and bolus-infusion protocols we ceased infusion at the 55-min mark. We expected radioactivity to remain stable for a short time afterwards. However, both protocols showed a clear decline in radioactivity when infusion ceased.

In sum, on the basis of the plasma radioactivity curves alone, it is apparent that the bolus-infusion protocol provides the most stable signal over the course of the scan, which is maintained as long as infusion is administered.

### Grey matter signal

Consistent with the plasma radioactivity results, the grey matter mean signal increased fastest for bolus administration, followed by bolus-infusion, with the infusion protocol showing the slowest increase in signal (Fig. 2B). By the end of the experiment, 4 of the 5 bolus-infusion participants showed the highest signal intensity, with most (4 of 5) bolus participants showing a similar level of signal intensity to the infusion-only participants.

### fMRI results

**Figure 3:**
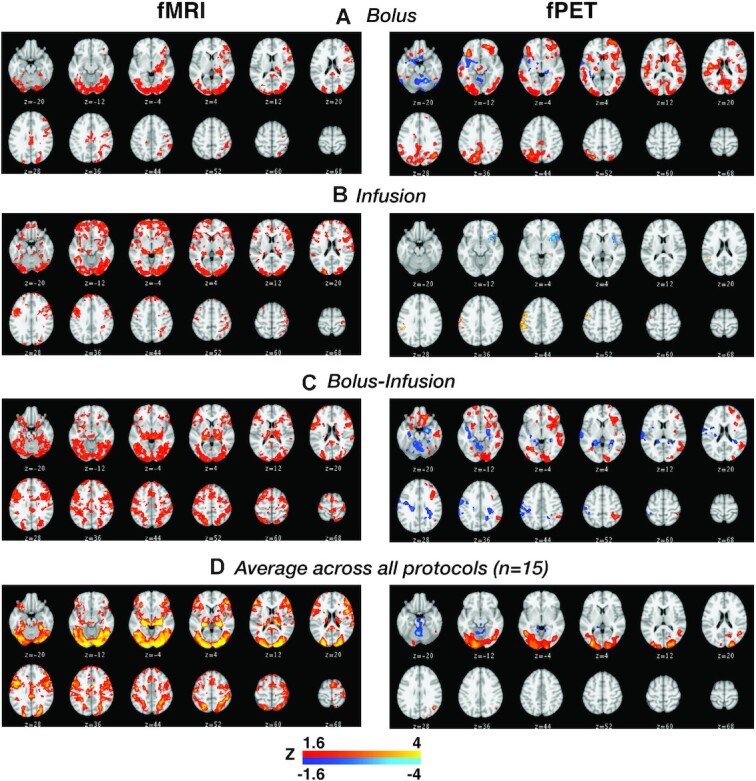
Group-level activation maps for task (Zcorr > 1.6) for (left) fMRI and (right) fPET; shown separately for **(A)** bolus group, **(B)** infusion group, **(C)** bolus-infusion group. Given that the fMRI protocol did not differ for the 3 groups we also show the group average fMRI across all 15 participants in panel **D**.

The fMRI results are shown primarily to confirm that the experimental design was successful in eliciting stimulus-evoked fMRI responses in the visual cortex (Fig. 3). As expected, visual cortex was active for all 3 groups (and in the average across the 15 participants; Fig. 3D); additional activity was also apparent in other cortical areas known to be involved in processing visual stimuli, including the intraparietal sulcus and frontal eye fields.

### fPET results

Across the 3 protocols (Fig. 3) task-related fPET showed a more focal pattern of activity in the visual cortex compared to fMRI. Visual comparison of the 3 administration protocols showed only modest levels of activity in the infusion-only protocol (Fig. 3B), with more widespread cortical activity in the bolus-only (Fig. 3A) and bolus-infusion protocols (Fig. 3C). The bolus-infusion protocol showed more widespread “negative” uptake than the other administration protocols, suggesting that these regions showed slower uptake of FDG by comparison to the grey matter mean.

**Figure 4:**
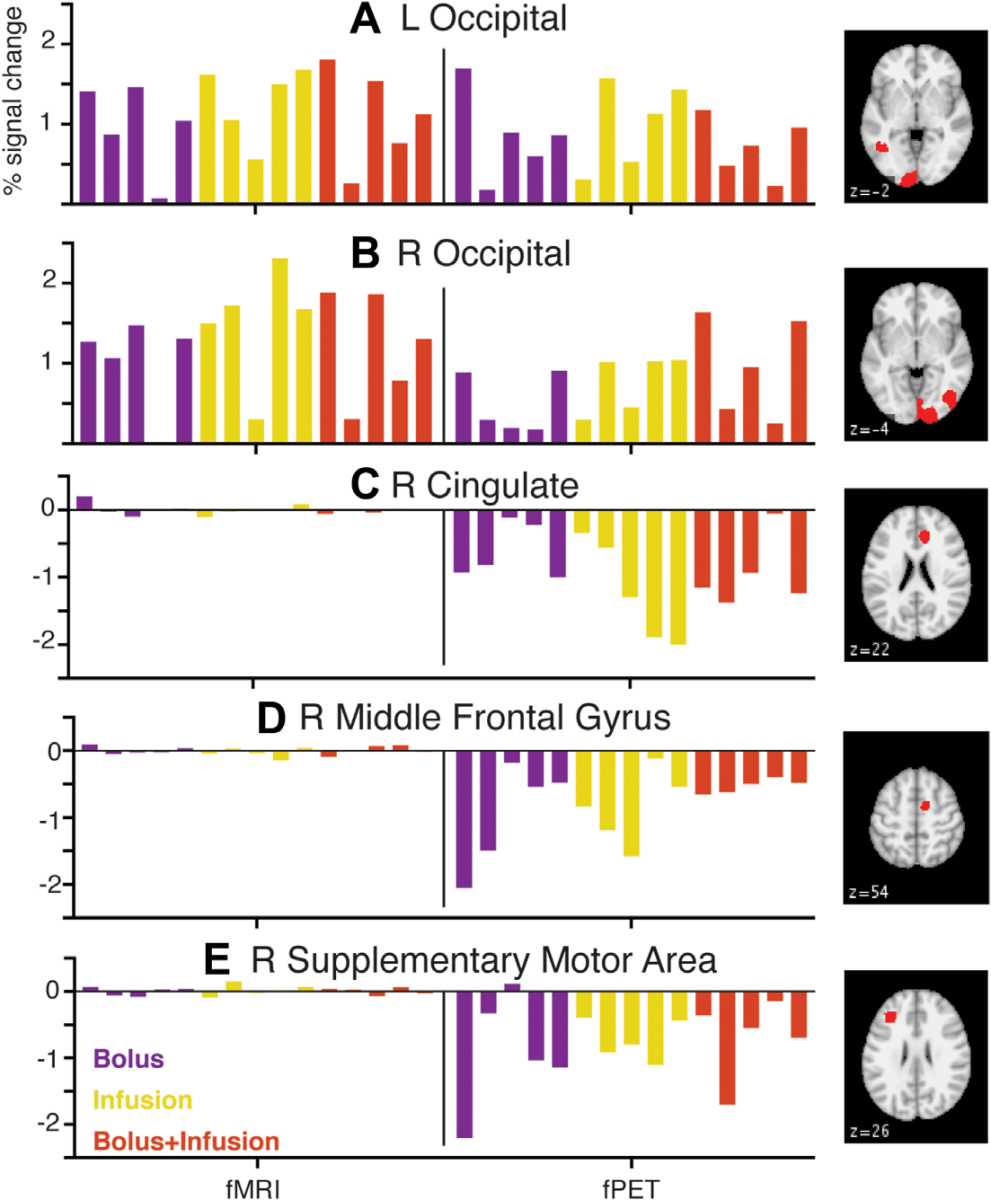
Percent signal change for 5 regions of interest for each administration method. Each column represents a single participant. Percent signal change is calculated as the β-regressor for all task blocks relative to rest blocks. L: left; R: right.

We visualized individual variability in percent signal change in 5 regions of interest for fMRI and fPET (Fig. 4). ROIs were defined as those that showed suprathreshold activity −2.3 > *z* > 2.3 in the middle blocks (Blocks 2, 3, 4) of the fPET data. Blocks 2, 3, 4 were chosen to coincide with the most stable activity across the 3 administration protocols. Figure 4 (right panels) shows the regions of interest. In the primary visual cortex (Fig. 4A and B), participants uniformly showed positive percent signal change for both the fMRI and fPET. In the frontal regions of interest, fPET showed a uniform negative percent signal change, suggesting slower uptake compared to grey matter; whereas fMRI showed close to zero percent signal change for all participants. It is notable that within each group (bolus, infusion, bolus-infusion) there is quite a bit of variability between individuals of 1–1.5% for both fMRI and fPET. Evaluating the fPET percent signal change, no single administration method seems to provide a more consistent fPET signal change across individuals; or a higher fPET signal change than the others.

**Figure 5:**
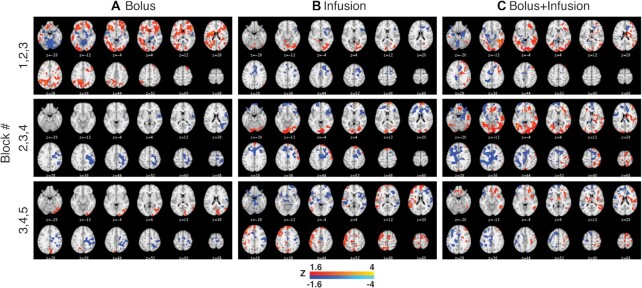
fPET results for Blocks 1, 2, 3 (top), 2, 3, 4 (middle), and 3, 4, 5 (bottom) for each administration protocol.

Finally, because Fig. 2 suggests that each administration protocol shows different timeframes for peak signal, we visualized fPET activity across 3 blocks at the start (Blocks 1, 2, 3), middle (Blocks 2, 3, 4), and end (Blocks 3, 4, 5) of the scan period (Fig. 5). The bolus-only protocol (Fig. 5A) showed the largest amount of suprathreshold activity at the start of the experiment (Blocks 1, 2, 3), with less activity in the middle and end of the experiment. While activity in the visual cortex is evident, there is substantial additional suprathreshold activity across the cortex. The infusion-only protocol (Fig. 5B) showed the smallest amount of suprathreshold activity across the blocks. Even though signal uptake is highest at the end of the experiment for this protocol (Fig. 2), there is little suprathreshold activity in the visual cortex evident during this period (Blocks 3, 4, 5). Suprathreshold visual cortex activity is evident in the middle blocks (2, 3, 4) for this protocol. The bolus-infusion protocol (Fig. 5C) showed the most sustained suprathreshold visual cortex activity compared to the bolus and infusion protocols; activity was evident in Blocks 1, 2, 3 and 2, 3, 4 but little activity in Blocks 3, 4, 5. Like the bolus-only group, the bolus-infusion group showed additional activity outside the visual cortex, which may represent false-positive activity.

## Concluding Remarks and Reuse Potential

Simultaneous MR-PET is a nascent technique, opening up many opportunities for scientific discovery, methods development, and signal optimization of dual-modality data. Although few imaging facilities worldwide currently possess the infrastructure and technical skill to acquire fPET-fMRI data, the rapid increase in publication (e.g., [5–7, 10–12, 15, 28, 40]) and reuse metrics of publicly available datasets [41, 42] attests to the value that the international neuroscience community places on this novel data type. The Monash DaCRA fPET-fMRI dataset is the only publicly available dataset that allows comparison of radiotracer administration protocols for fPET-fMRI. We provide both raw (listmode) and reconstructed fPET data to maximize the reuse value of the dataset. With listmode and reconstructed data, examples of reuse include the development of new processing pipelines and signal optimization methods that take into account variability in radiotracer dynamics related to differences in administration method. Release of listmode PET data is notable; to our knowledge only 1 other open source dataset includes listmode PET data: the Monash visfPET-fMRI dataset [28]. These data releases are very novel, occurring prior to the formalization of the PET BIDS standard (BEP009) [43, 44]. The draft PET BIDS standard does not yet extend to listmode data [44], so we applied our BIDS-like standard [28] to ensure that it is consistent with the interoperability principle of the FAIR philosophy. We [28] have previously demonstrated that listmode fPET data can be accurately reconstructed using open source methods STIR [45] and SIRF [46], confirming that the Monash DaCRA fPET-fMRI dataset is also consistent with the reusability principle of the FAIR philosophy. One caveat for the listmode data is that it is not possible to release the proprietary point-spread function (PSF) data for partial volume error (PVE) correction during reconstruction. We have previously compared non–PSF-corrected reconstructions using vendor-supplied and open source (SIRF) algorithms for open source listmode data without PSF information [28]. Alternatively, PVE correction using iterative deconvolution may also be applied to data where PSF information is unavailable [47].

Open source fPET-fMRI datasets provide many opportunities for progress in methods development: in the acquisition of the images, image reconstruction, data preparation, and analysis. The present dataset provides the opportunity to explore radiotracer dynamics of the constant-infusion administration approach; the Monash rsfPET-fMRI dataset [41] provides the opportunity to explore resting-state dynamics in healthy controls; and the Monash visfPET-fMRI dataset [42] provides the opportunity to explore responses to visual checkerboard stimulation with low-dose constant-infusion administration. The complementary nature of haemodynamic and “glucodynamic” responses to brain activity also presents an excellent opportunity for neuroscientific discovery. One example of reuse is to explore the differences between sexes in radiotracer uptake between the 3 groups [48]; differences in connectivity across blocks and time between the administration protocols; and another is to develop new signal optimization techniques that provide shorter frame durations than the provided 16-sec bins [5, 49]. One area where further development is required is in the development of accurate GLMs for the analysis of task-based responses. Standard practices exist for GLM analysis of fMRI data (e.g., SPM and FSL-based approaches), but these do not yet exist for fPET data. The Vienna group [5, 10, 11, 15] have reported a number of GLM-based analyses, which are analogous to block-design fMRI analyses. A number of questions remain: e.g., there is not yet agreement on the best way to manage the increasing baseline signal related to radiotracer dynamics over the course of the scan. Here we have presented 1 approach to GLM analysis of task-based fPET data; however more work is required to validate the approach. This dataset provides an excellent opportunity to develop task-based fPET analyses that account for underlying variability in radiotracer administration and uptake.

## Data Availability

All data supporting this work are openly available in the OpenNeuro database as dataset No. ds003397, under a CC0 public domain license [17].

## Abbreviations

BIDS: Brain imaging data structure; BOLD-fMRI/FDG-fPET: blood oxygenation level–dependent functional magnetic resonance imaging [^18^F]-fluorodeoxyglucose functional positron emission tomography; DaCRA: Dataset for Comparison of Radiotracer Administration; EPI: echo planar images; FDG: [^18^F]-fluorodeoxyglucose; FDG-*f*PET: [^18^F]-fluorodeoxyglucose functional positron emission tomography; FDG-PET: [^18^F]-fluorodeoxyglucose positron emission tomography; FLAIR: fluid attenuation inversion recovery; fMRI: functional magnetic resonance imaging; FOV: field of view; fPET: functional positron emission tomography; fPET-fMRI: simultaneous functional positron emission tomography–functional magnetic resonance imaging; FWHM: full width at half-maximum; GLM: general linear model; ICA: independent component analysis; JSON: Javascript Object Notation; MPRAGE: magnetization prepared rapid gradient echo; MRI: magnetic resonance imaging; OP-OSEM: ordinary Poisson ordered subset expectation maximization; P-A: posterior-anterior; PseudoCT: pseudo computed tomography; PVE: partial volume error; ROI: region of interest; SWI: susceptibility-weighted imaging; TA: acquisition time; TE: echo time; TR: repetition time; tsv: tab-separated values; UTE: ultrashort echo time.

## Consent for Publication

Consent was obtained from participants to release de-identified data.

## Competing Interests

Siemens Healthineers contributed financial support to the ARC Linkage Project held by G.F.E., S.D.J., and Z.C. The authors declare that they have no other competing interests.

## Funding

This work was supported by an Australian Research Council (ARC) Linkage Project (LP170100494) to PIs G.F.E., S.D.J., and Z.C. that includes financial support from Siemens Healthineers. S.D.J., P.G.D.W., and G.F.E. are supported by the ARC Centre of Excellence for Integrative Brain Function (CE140100007; PI: G.F.E.). S.D.J. is supported by an Australian National Health and Medical Research Council (NHMRC) Fellowship (APP1174164; PI: S.D.J.).

## Authors' Contributions

S.D.J.: Conceptualization, funding acquisition, investigation, methodology, project administration, resources, supervision, visualization, writing—original draft, writing—review and editing.

E.X.L.: Data curation, formal analysis, software, validation, visualization, writing—original draft.

S.Z.: Data curation, software, writing—review and editing.

P.G.D.W.: Conceptualization, formal analysis, investigation, methodology, supervision.

A.C.: Conceptualization, data curation, investigation, methodology.

R.M.: Data curation, investigation, methodology.

Z.C.: Funding acquisition, resources, supervision.

G.F.E.: Funding acquisition, resources, supervision, writing—review and editing.

## Supplementary Material

giac031_GIGA-D-21-00232_Original_Submission

giac031_GIGA-D-21-00232_Revision_1

giac031_GIGA-D-21-00232_Revision_2

giac031_Response_to_Reviewer_Comments_Original_Submission

giac031_Response_to_Reviewer_Comments_Revision_1

giac031_Reviewer_1_Report_Original_Submission Antoine Verger -- 9/8/2021 Reviewed

giac031_Reviewer_1_Report_Revision_1 Antoine Verger -- 2/4/2022 Reviewed

giac031_Reviewer_2_Report_Original_Submission Nicolas Costes -- 9/16/2021 Reviewed

giac031_Reviewer_3_Report_Original_Submission Chris Armit -- 10/11/2021 Reviewed
