# Supplementary material for: Monash DaCRA fPET-fMRI: A dataset for comparison of radiotracer administration for high temporal resolution functional FDG-PET
Source: Gigascience. 2022 Apr 30;11:giac031. doi: 10.1093/gigascience/giac031 (PMC9055854; doi:10.1093/gigascience/giac031)
Supplement: giac031_Response_to_Reviewer_Comments_Original_Submission [file giac031_response_to_reviewer_comments_original_submission.pdf]

Author's Response To Reviewer Comments

Close

Dear Prof Nogoy,

Thank you for inviting a minor revision of our manuscript. We respond to each Reviewers' Comments in detail in the attached Rejoinder.

With regards,
Sharna Jamadar

Close
