# Supplementary material for: Monash DaCRA fPET-fMRI: A dataset for comparison of radiotracer administration for high temporal resolution functional FDG-PET
Source: Gigascience. 2022 Apr 30;11:giac031. doi: 10.1093/gigascience/giac031 (PMC9055854; doi:10.1093/gigascience/giac031)
Supplement: giac031_Response_to_Reviewer_Comments_Revision_1 [file giac031_response_to_reviewer_comments_revision_1.pdf]

Author's Response To Reviewer Comments

Close

Dear Editor,

We respond to the Editor comments in full in the attached Rejoinder.

With regards
Sharna Jamadar

Close
