# Supplementary material for: Monash DaCRA fPET-fMRI: A dataset for comparison of radiotracer administration for high temporal resolution functional FDG-PET
Source: Gigascience. 2022 Apr 30;11:giac031. doi: 10.1093/gigascience/giac031 (PMC9055854; doi:10.1093/gigascience/giac031)
Supplement: giac031_Reviewer_1_Report_Original_Submission — Antoine Verger -- 9/8/2021 Reviewed [file giac031_reviewer_1_report_original_submission.pdf]

Reviewer Report

Title: Data Note: Monash DaCRA fPET-fMRI: A DAtaset for Comparison of Radiotracer Administration for high temporal resolution functional FDG-PET

Version: Original Submission **Date:** 9/8/2021

Reviewer name: Antoine Verger

Reviewer Comments to Author:

Review on "Data Note: Monash DaCRA fPET-fMRI: A DAtaset for Comparison of Radiotracer Administration for high temporal resolution functional FDG-PET"
This article is an important contribution in its field. This study is an open access dataset, Monash DaCRA fPET-fMRI, which contrasts three radiotracer administration protocols for FDG-fPET: bolus, constant infusion and hybrid bolus/infusion. The Monash DaCRA fPET-fMRI dataset is the only publicly available dataset that allows comparison of radiotracer administration protocols for fPET-fMRI. Even if the provided dataset is useful for the scientific community, the validation part needs some explanations.
Comments:
- Shame that this dataset is not available also for rest fPET-fMRI images. Indeed, most of the studies are also performed at rest (connectivity of neurodegenerative disorders for example) and should need some controls. Please discuss the opportunity to provide such databases.
- Was the administered FDG dose unique for all patients or adapted to the body weight? Please detail.
- The authors should discuss the gender variability across the 3 groups. Metabolism and radiotracer uptake is dependent of gender. The authors should at least include this covariate in their group analyses.
- Of course, raw data are available. I have nonetheless one question: what is the interest of using PSF and after a Gaussian filter in reconstructed images?
Why using PSF in dynamic PET (noisy) images? Please, can the authors justify the 16sec of frames for reconstruction of their images? Was it justified by any optimization?
- The authors further applied a filter of FWHM 12 mm after having previously reconstructed their images with a Gaussian filter? They should choose one of these two filters. If not, smoothing of PET images is too important.
- For the validation set at the group level, is the PET intensity normalization based on proportional scaling? It is particularly important to understand how the authors have obtained the grey matter mean signal.
- How was the grey matter mean signal obtained? From a grey matter MRI mask?
- Could the authors develop the way to have access to open access reconstructions algorithms? Particularly if images have been obtained with Biograph Siemens. They mention STIR and SIRF: please develop: is it able for anyone who has no access to a Siemens reconstruction algorithm? Is a specific PSF reconstruction for Siemens is implemented?
- "there has not yet is not yet agreement in the best way to manage" : please rephrase.
- Figure 1: Please include the conventional MRI sequences at the beginning of the acquisition.

- Figure 2: Please provide units for signal intensity? It would be also more comfortable to provide elements to distinguish the tasks from the rest periods.
- Figure 2: is the grey matter signal obtained for all the grey matter or only for the occipital cortex? Should the authors discuss the higher variability observed between patients for methods with bolus? Is it linked to the different sex ratio between the protocols? Discuss
Why one patient in the infusion protocol has a truncated time-activity curve?
- Figure 3: the authors should explain the variability of fMRI patterns in GLM albeit the same protocol was performed. Is there an influence of the coupled glycolytic metabolism?
- Figure 3: how the authors explain the absence of correlation with task in the infusion protocol? (this was not observed in the 3 phases of the protocols for infusion in Figure 5).
- Figure 4: define how the increase in signal percentage was calculated? How was the grey-matter normalized at the group level? Proportional scaling can be source of false positive abnormalities.
- Figure 5: Can the authors display the changes in connectivity of the occipital area between the 3 phases for each protocol? (by adding a supplemental part at the bottom of the Figure)

Level of Interest

Please indicate how interesting you found the manuscript: Choose an item.

Quality of Written English

Please indicate the quality of language in the manuscript: Choose an item.

Declaration of Competing Interests

Please complete a declaration of competing interests, considering the following questions:

- Have you in the past five years received reimbursements, fees, funding, or salary from an organisation that may in any way gain or lose financially from the publication of this manuscript, either now or in the future?
- Do you hold any stocks or shares in an organisation that may in any way gain or lose financially from the publication of this manuscript, either now or in the future?
- Do you hold or are you currently applying for any patents relating to the content of the manuscript?
- Have you received reimbursements, fees, funding, or salary from an organization that holds or has applied for patents relating to the content of the manuscript?
- Do you have any other financial competing interests?
- Do you have any non-financial competing interests in relation to this paper?

If you can answer no to all of the above, write 'I declare that I have no competing interests' below. If your reply is yes to any, please give details below.

I declare that I have no competing interests

I agree to the open peer review policy of the journal. I understand that my name will be included on my report to the authors and, if the manuscript is accepted for publication, my named report including any attachments I upload will be posted on the website along with the authors' responses. I agree for my report to be made available under an Open Access Creative Commons CC-BY license (http://creativecommons.org/licenses/by/4.0/). I understand that any comments which I do not wish to be included in my named report can be included as confidential comments to the editors, which will not be published.

Choose an item.

To further support our reviewers, we have joined with Publons, where you can gain additional credit to further highlight your hard work (see: https://publons.com/journal/530/gigascience). On publication of this paper, your review will be automatically added to Publons, you can then choose whether or not to claim your Publons credit. I understand this statement.

Yes Choose an item.
