# Supplementary material for: Monash DaCRA fPET-fMRI: A dataset for comparison of radiotracer administration for high temporal resolution functional FDG-PET
Source: Gigascience. 2022 Apr 30;11:giac031. doi: 10.1093/gigascience/giac031 (PMC9055854; doi:10.1093/gigascience/giac031)
Supplement: giac031_Reviewer_2_Report_Original_Submission — Nicolas Costes -- 9/16/2021 Reviewed [file giac031_reviewer_2_report_original_submission.pdf]

Reviewer Report

Title: Data Note: Monash DaCRA fPET-fMRI: A DAtaset for Comparison of Radiotracer Administration for high temporal resolution functional FDG-PET

Version: Original Submission **Date:** 9/16/2021

Reviewer name: Nicolas Costes

Reviewer Comments to Author:

Jadamar et al present a database of limited size, but of a rarity which amply justifies its interest. This is a combined dynamic FDG PET (fTEP) and fMRI study performed in three groups of 5 subjects for whom 3 different modes of FDG administration were used: bolus, infusion and bolus + infusion. The statistical analysis resulting from this study is also of limited scope due to the low residual degree of freedom of the design, but nevertheless makes it possible to confirm the expected characteristics of the shape of PET kinetics; It confirms the superiority of the bolus + infusion protocol ensuring maximum sensitivity to highlighting the neural circuits involved in the visual flickering task performed during acquisition.
The interest of the study lies in the free provision of the whole data that can be used, as it is argued, as a demonstrator for the development of methods for correcting, processing and analyzing data. A multivariate analysis combing PET and fMRI taking advantage of the simultaneous recording is not accired out: a simple GLM voxel-to-voxel analysis makes it possible to expose notable differences between the 3 methods of administration of FDG. However, the provision of data opens the field for future exploitation. The fact that raw data before PET reconstruction is provided is relatively new and opens up the possibility of extending the field of their exploitation to methods of correction and reconstruction. Respecting the BIDS description format as much as possible is also a plus.
These data are of undeniable interest to the community and therefore the description of their content and the exhaustive provision of all the demographic and physical parameters of their realization deserve their publication.
Some following remarks should be considered before publication.
p7. [18F]-FDG 18 should be in upper script
p9: raw PE data are in the original format exported from the siemens console: is there a distinction between list-mode file exceeding 4 Gb, as it is the case on the Siemens console? In which format the raw data will be provided?
Results:
Figure 2: A. Please specify if plasma curves are corrected for 18F radioactivity decay at the time of injection.
Figure 3. Why was the correction applied for Zcorr? FWE? FDR?
Figure 4. How exactly Â« percent final change Â» is computed: is it an average of the active periods compared to rest period? Is it computed from the beta regressor or directly on signal change? In the later case, on which interval?
Figure 5. A well the average accros all protocols is provided in Fig3.D to serve as a reference, could you also provide the average accros

References

Please review references: check for incomplete references (2., 8., 21. for example), uniformity of format and provide DOI as it is already done for the majority of your them.

Level of Interest

Please indicate how interesting you found the manuscript: Choose an item.

Quality of Written English

Please indicate the quality of language in the manuscript: Choose an item.

Declaration of Competing Interests

Please complete a declaration of competing interests, considering the following questions:

- Have you in the past five years received reimbursements, fees, funding, or salary from an organisation that may in any way gain or lose financially from the publication of this manuscript, either now or in the future?
- Do you hold any stocks or shares in an organisation that may in any way gain or lose financially from the publication of this manuscript, either now or in the future?
- Do you hold or are you currently applying for any patents relating to the content of the manuscript?
- Have you received reimbursements, fees, funding, or salary from an organization that holds or has applied for patents relating to the content of the manuscript?
- Do you have any other financial competing interests?
- Do you have any non-financial competing interests in relation to this paper?

If you can answer no to all of the above, write 'I declare that I have no competing interests' below. If your reply is yes to any, please give details below.

I declare that I have no competing interests'

I agree to the open peer review policy of the journal. I understand that my name will be included on my report to the authors and, if the manuscript is accepted for publication, my named report including any attachments I upload will be posted on the website along with the authors' responses. I agree for my report to be made available under an Open Access Creative Commons CC-BY license (http://creativecommons.org/licenses/by/4.0/). I understand that any comments which I do not wish to be included in my named report can be included as confidential comments to the editors, which will not be published.

Choose an item.

To further support our reviewers, we have joined with Publons, where you can gain additional credit to further highlight your hard work (see: https://publons.com/journal/530/gigascience). On publication of this paper, your review will be automatically added to Publons, you can then choose whether or not to claim your Publons credit. I understand this statement.

Yes Choose an item.
