# Supplementary material for: Monash DaCRA fPET-fMRI: A dataset for comparison of radiotracer administration for high temporal resolution functional FDG-PET
Source: Gigascience. 2022 Apr 30;11:giac031. doi: 10.1093/gigascience/giac031 (PMC9055854; doi:10.1093/gigascience/giac031)
Supplement: giac031_Reviewer_3_Report_Original_Submission — Chris Armit -- 10/11/2021 Reviewed [file giac031_reviewer_3_report_original_submission.pdf]

Reviewer Report

Title: Data Note: Monash DaCRA fPET-fMRI: A DAtaset for Comparison of Radiotracer Administration for high temporal resolution functional FDG-PET

Version: Original Submission **Date:** 10/11/2021

Reviewer name: Chris Armit

Reviewer Comments to Author:

This Data Note describes an Open CC0 neuroimaging dataset of 15 subjects (young adults) who underwent simultaneous BOLD-fMRI and FDG-fPET imaging. FDG-fPET ([18]-fluorodeoxyglucose positron emission tomography) measures glucose uptake in the human brain, whereas BOLD-fMRI (blood oxygenation level dependent functional magnetic resonance imaging) captures the cerebrovascular haemodynamic response. FDG-PET data was acquired using three different radiotracer administration protocols - bolus, constant infusion, and 50% bolus + 50% infusion - and each administration protocol was applied to 5 subjects. BOLD-fMRI and FDG-PET was acquired while participants viewed a checkerboard stimulation, which was used to trigger dynamic changes in brain glucose metabolism.
This neuroimaging dataset allows researchers to explore the complexity of energetic dynamics in the brain using multimodal imaging data analysis. In addition, this neuroimaging dataset includes structural MRI data for each of the subject, including T1 and T2 FLAIR, enabling neuroanatomical correlations to be explored.
The neuroimaging data are available from OpenNeuro [http://doi.org/10.18112/openneuro.ds003397.v1.1.1] and the authors are to be commended for ascribing a CC0 Public Domain Dedication to this dataset. Importantly, the authors highlight that consent was obtained from participants to release de-identified data. I downloaded a small number of image files from this dataset and I confirm that the de-identified NIfTI (Neuroimaging Informatics Technology Initiative) format files can be opened using Fiji / ImageJ.
This neuroimaging dataset has immense reuse potential and I recommend this Data Note for publication in GigaScience.

Level of Interest

Please indicate how interesting you found the manuscript: Choose an item.

Quality of Written English

Please indicate the quality of language in the manuscript: Choose an item.

Declaration of Competing Interests

Please complete a declaration of competing interests, considering the following questions:

- Have you in the past five years received reimbursements, fees, funding, or salary from an organisation that may in any way gain or lose financially from the publication of this manuscript, either now or in the future?
- Do you hold any stocks or shares in an organisation that may in any way gain or lose financially from the publication of this manuscript, either now or in the future?
- Do you hold or are you currently applying for any patents relating to the content of the manuscript?
- Have you received reimbursements, fees, funding, or salary from an organization that holds or has applied for patents relating to the content of the manuscript?
- Do you have any other financial competing interests?
- Do you have any non-financial competing interests in relation to this paper?

If you can answer no to all of the above, write 'I declare that I have no competing interests' below. If your reply is yes to any, please give details below.

I work for GigaScience where I perform checks on the quality of image data and the image analysis approach. My funding is not dependent on the outcome of this review, and I declare that I have no competing interests.

I agree to the open peer review policy of the journal. I understand that my name will be included on my report to the authors and, if the manuscript is accepted for publication, my named report including any attachments I upload will be posted on the website along with the authors' responses. I agree for my report to be made available under an Open Access Creative Commons CC-BY license (http://creativecommons.org/licenses/by/4.0/). I understand that any comments which I do not wish to be included in my named report can be included as confidential comments to the editors, which will not be published.

Choose an item.

To further support our reviewers, we have joined with Publons, where you can gain additional credit to further highlight your hard work (see: https://publons.com/journal/530/gigascience). On publication of this paper, your review will be automatically added to Publons, you can then choose whether or not to claim your Publons credit. I understand this statement.

Yes Choose an item.
